# Homologous Expression of the *Caldicellulosiruptor bescii* CelA Reveals that the Extracellular Protein Is Glycosylated

**DOI:** 10.1371/journal.pone.0119508

**Published:** 2015-03-23

**Authors:** Daehwan Chung, Jenna Young, Yannick J. Bomble, Todd A. Vander Wall, Joseph Groom, Michael E. Himmel, Janet Westpheling

**Affiliations:** 1 Department of Genetics, University of Georgia, Athens, Georgia, United States of America; 2 Biosciences Center, National Renewable Energy Laboratory, Golden, Colorado, United States of America; 3 The BioEnergy Science Center, Oak Ridge National Laboratory, Oak Ridge, Tennessee, United States of America; Weizmann Institute of Science, ISRAEL

## Abstract

Members of the bacterial genus *Caldicellulosiruptor* are the most thermophilic cellulolytic microbes described with ability to digest lignocellulosic biomass without conventional pretreatment. The cellulolytic ability of different species varies dramatically and correlates with the presence of the multimodular cellulase CelA, which contains both a glycoside hydrolase family 9 endoglucanase and a glycoside hydrolase family 48 exoglucanase known to be synergistic in their activity, connected by three cellulose-binding domains via linker peptides. This architecture exploits the cellulose surface ablation driven by its general cellulase processivity as well as excavates cavities into the surface of the substrate, revealing a novel paradigm for cellulase activity. We recently reported that a deletion of *celA* in *C*. *bescii* had a significant effect on its ability to utilize complex biomass. To analyze the structure and function of CelA and its role in biomass deconstruction, we constructed a new expression vector for *C*. *bescii* and were able, for the first time, to express significant quantities of full-length protein *in vivo* in the native host. The protein, which contains a Histidine tag, was active and excreted from the cell. Expression of CelA protein with and without its signal sequence allowed comparison of protein retained intracellularly to protein transported extracellularly. Analysis of protein in culture supernatants revealed that the extracellular CelA protein is glycosylated whereas the intracellular CelA is not, suggesting that either protein transport is required for this post-translational modification or that glycosylation is required for protein export. The mechanism and role of protein glycosylation in bacteria is poorly understood and the ability to express CelA *in vivo* in *C*. *bescii* will allow the study of the mechanism of protein glycosylation in this thermophile. It will also allow the study of glycosylation of CelA itself and its role in the structure and function of this important enzyme in biomass deconstruction.

## Introduction

CelA has been the recent focus of intense study because of its remarkable activity. CelA alone is more active than the key enzymes found in commercial enzyme mixtures used for biomass pretreatment and has an activity similar to the entire purified *C*. *bescii* secretome [1]. CelA contains two enzymatic domains with synergistic activities, a GH family-9 endoglucanase and a GH family 48 exoglucanase. However, study of CelA *in vitro* has been limited by the inability to express and purify the full-length protein in sufficient quantities.

CelA is the most abundant extracellular protein produced by *C*. *bescii*, however, it is cleaved in culture supernatants [1, 2], making the isolation of large quantities of full-length protein difficult. Moreover, CelA heterologously expressed in *E*. *coli* is proteolytically degraded making isolation of full-length product virtually impossible [2, 3]. Full-length protein, as well as several truncated products, have been produced in *Bacillus megaterium* [3], but the protein produced differed substantially in molecular weight (MW) from that produced by the native *C*. *bescii* host. Interestingly, the observed molecular mass of CelA purified from *C*. *bescii* culture supernatants is 230 kDa—significantly larger than the predicted MW of 190 kDa [2]. There is one report that CelA blotted onto nitrocellulose stained positively using a glycoprotein detection kit [2] which might provide an explanation for the difference in observed MW, but no details of this experiment were provided. Taken together, previous work clearly indicates posttranslational modification of CelA *in vivo* and suggests that protein glycosylation may be involved.

While very little is known about the role of protein glycosylation in bacteria, it has been implicated in increased activity, thermostability, resistance to proteolytic cleavage, and binding affinity [4–9]. Many carbohydrate-active enzymes from filamentous fungi contain both *N*- and *O*-linked glycans; the extent and heterogeneity of glycosylation depends on the growth conditions, expression host, and presence of glycan trimming enzymes within the secretome [10]. Both *N*- and *O*-linked glycosylation are also known to exist in bacteria [11–13]. *N*-linked glycans result from covalent attachment to exposed surface asparagine resides. *O*-glycans are typically attached to exposed serine and threonine residues [11, 12]. The best understood examples of glycosylation in bacteria are associated with pathogen physiology, including the *O*-glycosylation of lipopolysaccharides (LPS) and pili, as well as *N*-glycosylation of flagella [14]. Very little is understood about the role of glycosylation in enzyme function and reports tend to be varied and somewhat contradictory. Comparison of endo- and exoglucanases expressed in *Cellulomonas fimi*, which are glycosylated, differ from their counterparts expressed in *E*. *coli* (non-glycosylated) and in this case the difference in glycosylation shows no difference in activity, stability to pH and temperature, or substrate binding. The major difference observed was in susceptibility to proteolysis with the glycosylated version remaining intact and the non-glycosylated form sensitive to cleavage [4]. Expression of non-glycosylated *Bacillus* enzymes in yeast, where they were heavily glycosylated, resulted in more thermostable versions [15]. The extent of glycosylation can also add to the complexity of the role of glycosylation. Increasing glycosylation of a *Trichoderma reesei* enzyme, Cel7A, by expressing it in *Aspergillus niger* reduced cellulolytic activity, but also resulted in increased binding affinity [8]. One can conclude that the alteration of individual residues associated with glycosylation can affect both activity and thermostability to varying degrees [7].

Here, we describe a new expression vector for high level, tagged protein expression in *C*. *bescii* and its use for homologous expression of full-length CelA protein in its native host. We provide direct evidence that extracellular CelA protein is glycosylated using an enhanced Periodic Acid-Schiff (PAS) method for detection of glycoprotein sugars. Confirmation that the glycosylated protein is, in fact, CelA comes from the fact that the protein was not present in a CelA deletion mutant and analysis of purified His-tagged protein by both reaction with anti-His-tag antibody and LC-MS/MS proteomic analysis. The ability to study CelA in its native form will allow the analysis of the mechanism of protein glycosylation in bacteria, as well as the impact of glycosylation on the structure and function of this important cellulase for biomass deconstruction.

## Results and Discussion

### Construction of an improved vector for expression of full-length CelA protein in *C*. *bescii*

**Fig 1:**
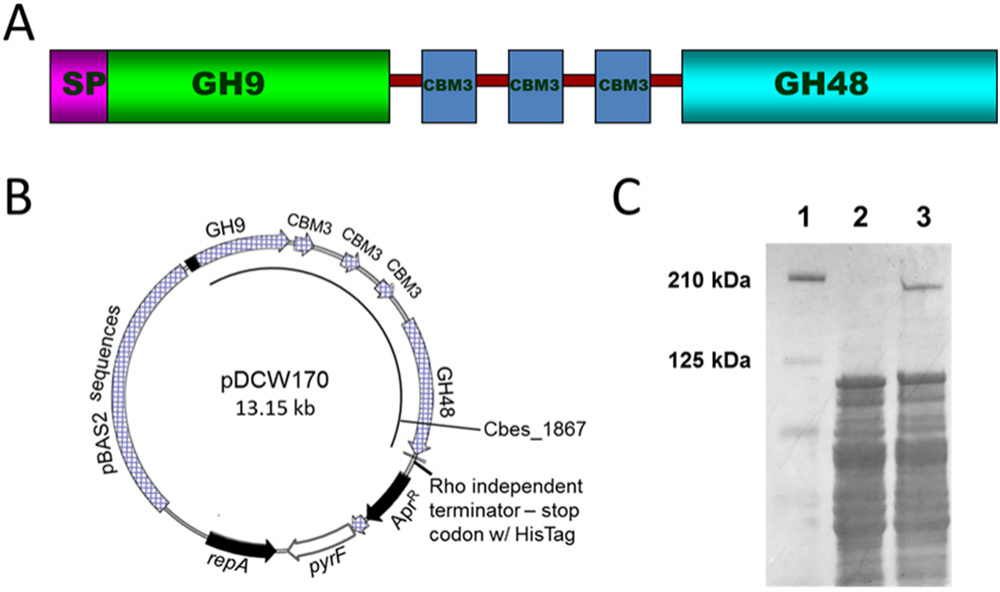
Expression of full-length CelA in *C*. *bescii*. A) A diagram of CelA: SP, signal peptide; GH9, Family 9A glycoside hydrolase domain; CBM, carbohydrate binding modules—one family 3 type C followed by two family 3 type B; GH48, Family 48 glycoside hydrolase domain. B). An expression cassette that contains the regulatory region of the *C*. *bescii* S-layer protein, a *C*-terminal 6X His-tag version of *celA* (Cbes1867), a Rho-independent terminator, the *pyrF* (from *C*. *thermocellum*) cassette for selection in *C*. *bescii* and pBAS2 sequences for replication in *Caldicellulosiruptor spp*. C) SDS-PAGE gel (4 to 15% gradient) stained with Coomassie Brilliant Blue showing 100 μg of cell free extract from: Lane 1, MW standards (BioRad); Lane 2, wild type *C*. *bescii* JWCB001; Lane 3, JWCB040 *ΔpyrFA* pDCW170::*celA*.

Previous attempts to express full-length CelA protein in *E*. *coli* were unsuccessful [2, 3] resulting in extensive proteolytic cleavage and low levels of expression, the latter likely due to codon usage bias of the heterologously expressed protein. Recently, full-length CelA and a truncated form of the protein including all three CBMs and the GH48 catalytic domain were successfully expressed in *Bacillus megaterium*; however, the protein produced was of the MW predicted from its sequence and did not indicate post-translational modification in this host [3]. A diagram of the domain architecture of CelA is shown in Fig. 1A. The polypeptide contains a signal peptide sequence for export and the signal peptidase cleavage site is predicted to be located following either residue 21 or 23 [1, 3]. Anticipating that expression of intracellular protein would reduce or eliminate issues with proteolysis, an expression vector, pDCW170, was constructed that contained the CelA protein without the signal sequence (Fig. 1B and Figure A in S1 File). This vector contains an apramycin resistant gene cassette (Apr^R^) for selection in *E*. *coli*, as well as a low copy replication origin (*pSC101*), a plasmid-encoded gene required for *pSC101* replication (*repA*), and a partition locus (*par*)—all of which are necessary for replication in *E*. *coli*. pDCW170 also contains a *pyrF* expression cassette for selection in *C*. *bescii* with the wild type *C*. *thermocellum pyrF* gene and pBAS2 sequences that include an origin of replication for *C*. *bescii*. Transcription of CelA is directed by the regulatory and *rho* independent terminator sequences surrounding Cbes2303 (S-layer protein) with a *C*-terminal 6X histidine-tag (Fig. 1B and Figure A in S1 File), followed by a stop codon. This expression cassette was recently successfully used for AdhE expression in *C*. *bescii* to produce bio-ethanol [16].

Plasmid DNA was transformed into a *pyrFA* deletion of *C*. *bescii* [17] and transformants were selected for uracil prototrophy. The CelA expression strain was grown at 65°C to accommodate expression of the *C*. *thermocellum pyrF* gene used for complementation and plasmid selection. The presence of the plasmid in transformants was confirmed by PCR analysis. Primers (DC228 and DC460) were used to amplify the portion of the plasmid containing the open reading frame of CelA, but annealing to regions of the plasmid outside the gene to avoid amplification of sequences residing on the chromosome. Amplicons were generated that confirmed transformation. Furthermore, a DNA fragment of ~5.7 kb confirmed the presence of the gene encoding CelA within the plasmid (Figure B in S1 File).

As shown in Fig. 1C, the CelA protein without its signal peptide was clearly detected in cell free extracts of the CelA expression strain, but not cell free extracts of the wild type strain. Importantly, the protein produced was full-length, and the MW was consistent with that predicted from amino acid sequence [2] and similar to CelA heterologously expressed in *B*. *megaterium* [3].

### Evidence that the extracellular form of the CelA protein in *C*. *bescii* is glycosylated

**Fig 2:**
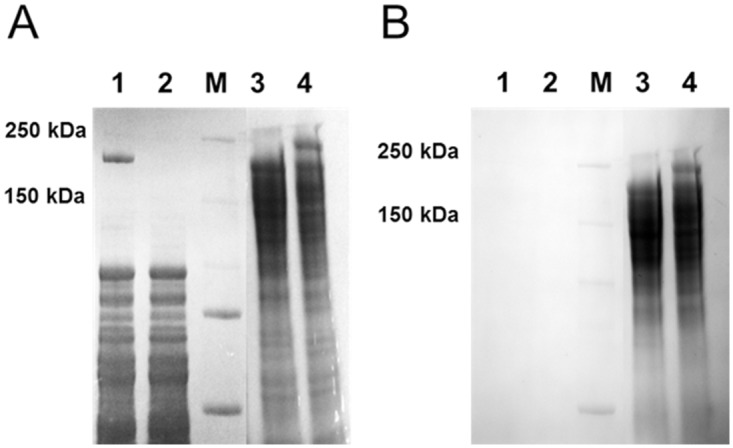
Evidence for extracellular glycosylation of CelA. Panel A, Coomassie stained gel. Panel B, the same gel stained with Glycoprotein Stain (G-Biosciences). Lane1, cell free extract from JWCB040 *ΔpyrFA* pDCW170::*celA* (no signal sequence); Lane 2, cell free extract from JWCB046 *ΔpyrFA ldh*::*ISCbe4 Δcbe1 ΔcelA* pDCW173::*celA* (with the signal sequence); M, Molecular weight markers (Biorad); Lane 3, supernatant from JWCB029 *ΔpyrFA ldh*::*ISCbe4 Δcbe1 ΔcelA*; Lane 4, JWCB046 *ΔpyrFA ldh*::*ISCbe4 Δcbe1 ΔcelA* pDCW173::*celA* (with the signal sequence).

Since intracellular expression of CelA showed no indication of post-translational modification, an expression vector with the signal sequence was constructed (Figure C in S1 File). This vector, pDCW173, is identical to the vector used for intracellular expression, except for the presence of the signal sequence. This plasmid DNA was transformed into the CelA deletion strain for analysis. As shown in Fig. 2A, abundant full-length CelA protein was produced in the strain containing the expression vector. Intracellular protein in cell free extract (CFE), as well as extracellular protein (ECP) in culture supernatants, was readily detected by SDS-PAGE analysis with Coomassie Brilliant Blue staining. No full-length CelA protein was detected in either the cell free extract from wild type or parent strain (Figure D in S1 File). Nor was full-length protein detected in the CFE from the strain containing the expression vector with the signal peptide. As had been previously observed [2, 3], the intracellular and extracellular forms of CelA differed in apparent MW, 190 kDa and 230 kDa, respectively. The signal sequence is 34 amino acids and accounts for ~3.7 kDa. An additional six amino acids are included in the vector to add the His-tag, adding another 0.66 kDa to the protein, however, this addition could not account for the difference in MW observed. The apparent difference between the two forms of CelA is approximately 40 kDa. As shown in Fig. 2B, the same gel was stained with the G-Biosciences Glycoprotein Staining Kit. The kit uses an enhanced Periodic Acid-Schiff (PAS) method for detection of glycoprotein sugars. An oxidizing agent first oxidizes the cis-diol sugar groups to aldehydes and the aldehyde groups react with the Glyco-Stain Solution forming Schiff bonds producing a strong magenta color. Only extracellular CelA protein reacted with this stain. To confirm that the band in question is actually CelA, the same reaction was performed on supernatants from a *C*. *bescii* strain containing a deletion of the *celA* gene [18]. No band corresponding to glycosylated CelA was present in this strain. In addition, full length CelA was purified using the added His-tag, and the purified protein was confirmed to be CelA by its reaction with an antibody to the His-tag and proteomic analysis using LC-MS/MS (Figure E in S1 File). Bioinformatic analyses were performed to predict potential glycosylation sites in the *C*. *bescii* CelA protein sequence using the GlycoPP webserver [19]. We identified six *N*-linked glycosylation sites in the GH9 catalytic domain (at positions 38, 237, 258, 280, 326, and 447) and five in the GH48 catalytic domain (at positions 1250, 1339, 1460, 1470, and 1578) using the Binary Profile of Pattern (BPP) method. *O*-linked glycosylation sites are predicted primarily in the CBMs and linker regions using the Position Specific Scoring Matrix (PSSM) profile.

While we have no direct evidence for the location of glycosylation, these results suggest that glycosylation is either required for protein export or that glycosylation occurs during protein export and export is required for post translational modification. Very little is known about the role of protein glycosylation in bacteria, and what has been reported is often contradictory. Most work has been done on the characterization of eukaryotic glycosylated proteins, and many fungal cellulases are known to have both *N*- and *O*-linked glycosylation. Changes in *N*-glycosylation of the catalytic domain of *T*. *reesei* Cel7A were shown experimentally to affect the activity and binding of the cellulase on crystalline cellulose [7], and recently, the role of the *O*-linked glycosylation of the linker regions of *T*. *reesei* Cel7A on cellulose binding was predicted by molecular dynamics simulation [20]. These results suggest that the general glycosylation of Cel7A greatly affects enzyme activity, as well as other properties, including thermal tolerance and protection from proteolytic cleavage [21]. Similar properties may be conferred by glycosylation of CelA. A close inspection of the full atomistic structure of CelA using a method similar to that found in Sammond *et al* [22], reveals that the linkers and also the catalytic and binding domains of CelA contain extensive hydrophobic patches. These patches appear to be in close proximity to the glycosylation sites predicted by GlycoPP for both N- and O-glycosylation patterns. One possibility is that glycosylation of CelA serves to modify its quaternary structure and limit the amount of aggregation when CelA is not productively bound to biomass.

Secretion in Gram-positive bacteria is thought to follow the classical protein secretion pathway as studied in *E*. *coli* [23, 24]. The *C*. *bescii* genome, specifically, includes genes annotated as *secA*, *secD*, *secE*, *secF*, *secG*, *secY*, *ftsY*, and *prsA*. Of particular interest are the *secA* and *secY* genes that exhibit homology to *secA2* and *secY2* that were shown to be necessary for the secretion of large glycoproteins [25–27]. A query of the *C*. *bescii* genome using GenBank [28] for *secA2* and *secY2* amino acid sequences derived from *S*. *gordonii* resulted in the identification of two proteins with sequence homology to SecA2 (Cbes2104; 98% of Sequence Query cover and 42% sequence identity) and SecY2 (Cbes1726; 87% of Sequence Query cover and 27% sequence identity). If these proteins are involved in secretion of glycosylated CelA protein, deletion of the genes that encode them should result in intracellular retention of the glycosylated protein. We are currently testing that prediction.

### Activity of homologously expressed CelA protein

**Fig 3:**
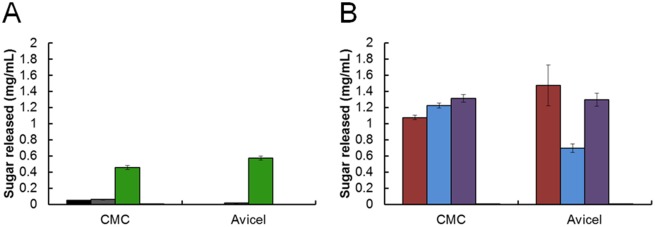
Enzymatic activity of full-length CelA produced intra- and extracellularly. Substrates used as sole carbon source were carboxymethylcellulose (CMC) and Avicel. A) Activity of intracellular (400 μg/mL CFE) protein fractions from the wild type JWCB001 (black), JWCB005 *ΔpyrFA* (grey), and JWCB040 *ΔpyrFA* pDCW170::*celA* (no signal sequence) (green). B) Culture supernatant (25 μg/mL of concentrated protein) from JWCB018 *ΔpyrFA ldh*::*ISCbe4 Δcbe1* (red), JWCB029 *ΔpyrFA ldh*::*ISCbe4 Δcbe1 ΔcelA* (blue), and JWCB046 *ΔpyrFA ldh*::*ISCbe4 Δcbe1 ΔcelA* pDCW173::*celA* (with the signal sequence) (purple).

To test whether the full-length, His-tagged CelA protein was active and to compare intracellular and extracellular enzyme activity, enzyme assays were performed using CMC and Avicel as substrates. CMC is a soluble form of cellulose used for estimating endoglucanase activity. Avicel, a microcrystalline form of cellulose, is primarily used to estimate exoglucanase activity. Because CelA is a bifunctional enzyme with endoglucanase and exoglucanase activities, both substrates were used to assay cellulolytic activity. We previously reported that the ECP fraction of the *celA* deletion strain showed a similar profile when acting on CMC to that of the wild type and parent strain whereas a reduction in enzyme activity was observed when acting on Avicel [18]. Please note that the figure in this previous report lists μg/ml in error (mg/ml is correct). For testing the intracellular protein, cell free extracts were prepared from the wild type strain, the parent strain for CelA expression, and the strain containing the CelA expression vector without the signal peptide (JWCB040). As shown in Fig. 3A, activity from the wild type and parent (JWCB005) strains on CMC or Avicel was detected, but at a very low levels. Extracts from the intracellular CelA expression strain released 0.46 (±0.02) mg/mL of glucose equivalents from CMC after one hour and 0.57 (±0.02) mg/mL of glucose equivalents from Avicel after 24 hours—significantly more than either of the background strains.

For extracellular CelA, expressed with the signal peptide, protein from JWCB018 (the parent strain for the *celA* deletion) and a *celA* deletion strain (JWCB029) were used for comparison with the strain containing the CelA expression vector incorporating the signal peptide (JWCB046). Culture supernatants were concentrated using a MW cutoff of 10 kDa followed by a buffer exchange with 20 mM MES and 2 mM 2-mercaptoethanol before comparing activity against CMC and Avicel. When tested on CMC, all three strains exhibited similar activity resulting in a release of 1.1 to 1.3 mg/mL of glucose equivalents. This result was similar to that previously reported [18] for JWCB018 and JWCB029. When tested on Avicel, a decrease in activity was observed for the CelA deletion (JWCB029) strain compared to the parent strain (JWCB018) (i.e., from 1.5 mg/mL to 0.67 mg/mL glucose equivalents released). Production of CelA from the expression strain restored activity on Avicel to wild-type levels (1.3 mg/mL glucose equivalents released) suggesting that the expression of CelA in this strain is substantial and the expression vector is working. We point out that the decrease in extracellular enzyme activity on Avicel in the CelA deletion strain was less than previously reported [18]. Protein used in those experiments was isolated from small batch, closed bottle cultures followed by protein precipitation. We now use protein isolated from buffer controlled fermenters followed by concentration with a 10 kDa molecular weight cutoff cartridge. The new protein preparation protocol, specifically concentrating the protein samples rather than precipitating the protein, results in higher enzyme activity.

## Conclusions

The newly constructed expression vectors, pDCW170 and pDCW173, were successfully used for both intra- and extracellular expression of active full-length CelA protein in its native host, *C*. *bescii* (Figs. 2 and 3). Homologous expression allowed detection of post-translational glycosylation of CelA protein, and this modification was only detected in extracellular protein. Confirmation that this protein was, in fact, CelA comes in part from the absence of the protein in the CelA deletion mutant; additionally, the purified His-tagged protein reacted with anti-His-tag antibody, and LC-MS/MS analysis confirmed the amino acid composition. Glycosylation has been shown to be one of the most important post-translational modification events to achieve full activity of multi-domain cellulases [8, 10, 29]. Since glycosylation has been shown to vary with heterologous expression [7, 8, 30, 31], the ability to produce CelA in its native host will be essential to understanding the role of glycosylation in terms of protein structure and function. This new ability to overexpress enzymes of interest in *C*. *bescii* will facilitate studies of protein engineering for the improvement of cellulolytic ability of this important thermophile.

## Methods

### Strains, media, and growth conditions

*Caldicellulosiruptor* and *E*. *coli* strains used in this study are listed in Table 1. *Caldicellulosiruptor* strains were grown anaerobically at 65°C on solid or in liquid low osmolarity defined (LOD) medium [32], as previously described, with maltose (0.5% w/v; catalog no. M5895, Sigma) or cellobiose as sole carbon source for routine growth and transformation experiments [33]. For growth of uracil auxotrophs JWCB005 (*ΔpyrFA*), JWCB018 (*ΔpyrFA ldh*::IS*Cbe4 Δcbe1*), and JWCB029 (*ΔpyrFA ldh*::IS*Cbe4 Δcbe1 ΔcelA*), the defined medium contained 40 μM uracil. This concentration of uracil does not support growth of *C*. *bescii* as sole carbon source. *E*. *coli* strain DH5α was used for plasmid DNA construction and preparation using standard techniques [34]. *E*. *coli* cells were cultured in LB broth supplemented with apramycin (50 μg/mL) and plasmid DNA was isolated using a Qiagen (Hilden, Germany) Miniprep kit.

**Table 1:** Strains and plasmids used in this work.

Strains/plasmids	Strain and genotype/phenotype	Source
*C*. *bescii*		
JWCB001	Wild type (*ura* ^+^/5-FOA^S^)	DSMZ^a^
JWCB005	*ΔpyrFA* (*ura* ^-^/5-FOA^R^)	[35]
JWCB018	*ΔpyrFA ldh*::*ISCbe4 Δcbe1* (*ura* ^-^/5-FOA^R^)	[33]
JWCB029	*ΔpyrFA ldh*::*ISCbe4 Δcbe1 ΔcelA* (*ura* ^-^/5-FOA^R^)	[18]
JWCB040	JWCB005 containing pDCW170 (*ura* ^+^/5-FOA^S^)	This study
JWCB046	JWCB029 containing pDCW173 (*ura* ^+^/5-FOA^S^)	This study
*Escherichia coli*		
JW332	DH5α containing pDCW170 (Apramycin^R^)	This study
JW335	DH5α containing pDCW173 (Apramycin^R^)	This study
Plasmids		
pJGW07	*E*. *coli*/*Caldicellulosiruptor* shuttle vector (Apramycin^R^)	[36]
pDCW170	Expression vector for intracellular CelA (Apramycin^R^)	This study
pDCW173	Expression vector for extracellular CelA (Apramycin^R^)	This study

^*a*^ *German Collection of Microorganisms and Cell Cultures*

Chromosomal DNA from *Caldicellulosiruptor* strains was extracted using the Quick-gDNA MiniPrep (Zymo, Irvine, CA) as previously described [37].

### Construction and transformation of CelA expression vectors

Plasmids used in this study were generated using high fidelity *pfu* AD DNA polymerase (Agilent Technologies, Santa Clara, CA), restriction enzymes (NEB, Ipswich, MA), and Fast-link DNA Ligase (Epicentre Technologies, Madison, WI) according to the manufacturer’s instructions. Plasmid pDCW170 (Fig. 1B, Figure A in S1 File) was constructed in two cloning steps. First, a 7.71 kb DNA fragment, containing the pSC101 replication origin for *E*. *coli*, a putative *C*. *bescii* replication origin, an apramycin resistance gene cassette (Apr^R^) and a *C*. *thermocellum pyrF* cassette, was amplified by PCR using primers DC481 (with PvuI site) and DC482 (with NotI site) using pJGW07 [36] as a template. The 3.28 kb DNA fragment, that contains 134 bp of upstream sequence of Cbes2303 (S-layer protein), 3,507 bp of Cbes2303 coding sequences, and 86 bp of its downstream sequence, was amplified using primers DC460 (with PvuI site) and DC461 (with NotI site) using *C*. *bescii* genomic DNA as a template. These two linear DNA fragments were digested with PvuI and NotI, and ligated to construct an 11.01 kb intermediate vector (Figure A in S1 File). In a second step, the 7.96 kb DNA fragment was amplified using primers DC464 (with BamHI site) and DC466 (with SphI site, 6X Histidine-tag, and stop codon) using the intermediate vector as template. A 5.181 kb DNA fragment containing the entire coding sequence of CelA (Cbes1867) except the 96 nucleotides encoding the signal peptide (Fig. 1A) was amplified by PCR using DC530 (with BamHI site and ATG) and DC371 (with SphI site) and *C*. *bescii* genomic DNA as template. These two linear DNA fragments were digested with BamHI and SphI, and ligated to construct a 13.15 kb plasmid, pDCW170 (Figure A in S1 File). Plasmid pDCW173 (Figure C in S1 File) is identical to pDCW170 except that it contains the entire coding sequence of Cbes1867 including the signal peptide. To make this change, a 5.277 kb DNA fragment containing the coding sequence of CelA was amplified by PCR using primers DC560 (with BamHI site) and DC371 (with SphI site) and *C*. *bescii* genomic DNA as template. *E*. *coli* strain DH5α cells were transformed by electroporation in a 2-mm-gap cuvette at 2.5 V and transformants were selected for apramycin resistance. The sequences of all plasmids were verified by Automatic sequencing (Genewiz, South Plainfield, NJ). All plasmids are available upon request.

Electrotransformation of cells was performed as previously described [38]. Cultures, electro-pulsed with plasmid DNA (~0.5 μg), were recovered in low osmolarity complex (LOC) growth medium [32] at 75°C. Recovery cultures were transferred to liquid LOD medium [32] without uracil to allow selection of uracil prototrophs. Cultures were plated on solid LOD media to obtain isolated colonies, and DNA was isolated from transformants. PCR amplification using primers (DC228 and DC460) outside the gene cassette on the plasmid was used to confirm the presence of the plasmid with the CelA gene present. Primers used for plasmid constructions and confirmation are listed in Table A in S1 File.

### Preparation of protein and detection of glycosylation

Cell free extracts (CFE) were prepared from 500 mL cells grown in closed bottles to mid-log phase at 65°C, harvested by centrifugation at 6,000 x g at 4°C for 15 min, resuspended in Cel-Lytic B cell lysis reagent (Sigma), and lysed by a combination of 4X freeze-thawing and sonication (3 to 4 times for 15 s at 40 amps with one min rests on ice). Samples were centrifuged to separate protein lysate from cell debris. Extracellular protein (ECP) was collected from 10 L cultures grown to mid-log phase at 65°C in a fermenter with pH control, centrifuged (8,000 x g at 4°C for 10 min), and filtered (glass fiber, 0.7 μm) to separate out cells, and concentrated with a 10 kDa molecular weight cut off column. The concentrated ECP was buffer exchanged with 20 mM MES/2 mM β-mercaptoethanol (pH 6.0), and protein concentrations were determined using the Bio-Rad protein assay kit with bovine serum albumin (BSA) as the standard. CFE and ECP (100 and 50 μg, respectively) were analyzed by SDS-PAGE using 4 to 15% gradient Mini-Protean TGX gels (Bio-Rad) run at 150 V for ~1.0 h. Proteins were visualized by staining with a Glycoprotein Staining Kit (G-Biosciences) according to the manufacturer’s instructions. After initial staining with the Glyco-stain solution, the SDS-PAGE gel was then stained with RAPIDstain solution to visualize all proteins.

### Enzyme activity assays

Cellulolytic activity was determined using 10 g/L of either CMC or Avicel in MES reaction buffer (pH 5.5) as previously described [39]. Four hundred μg/mL of intracellular CFE or 25 μg/mL extracellular protein was added to each reaction and incubated at 75°C (1 h for CMC and 24 h for Avicel). Controls were incubated for the same time without added enzyme. Reducing sugars in the supernatant were measured using dinitrosalicylic acid (DNS). Samples and standards (glucose) were mixed 1:1 with DNS and boiled for five min and measured at OD_575_. Activity was reported as mg/mL of sugar released.

## Supporting Information

S1 File Figure A, Construction of the intracellular version of full-length CelA expression vector pDCW170. Figure B, PCR confirmation of expression vector transformation. Figure C, Diagram of the version of full-length extracellular CelA expression vector pDCW173. Figure D, Evidence for extracellular glycosylation of CelA (expanded). Figure E, Purification and verification of extracellular His-tagged CelA. Table A, Primers used in this study. (DOCX)
