# Supplementary material for: Homologous Expression of the Caldicellulosiruptor bescii CelA Reveals that the Extracellular Protein Is Glycosylated
Source: PLoS One. 2015 Mar 23;10(3):e0119508. doi: 10.1371/journal.pone.0119508 (PMC4370642; doi:10.1371/journal.pone.0119508)
Supplement: S1 File — (DOCX) [file pone.0119508.s001.docx]

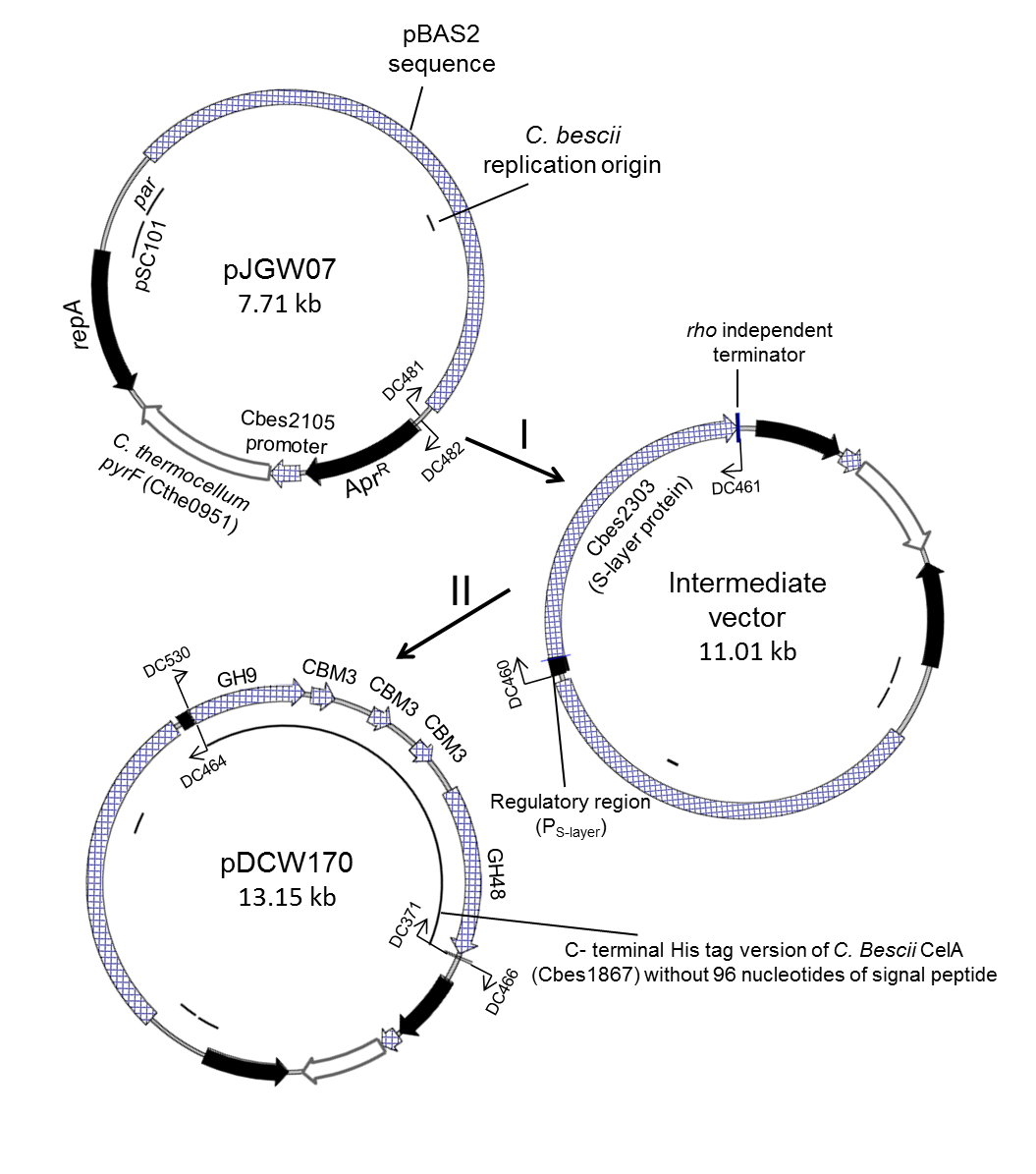


**Figure A. Construction of the intracellular version of full-length CelA expression vector pDCW170.** Plasmid pDCW170 was constructed in two cloning steps as described in the methods section. ORFs from *E. coli* are indicated as black arrows. As indicated: the apramycin resistant gene cassette (Apr^R^); *pSC101,* low copy replication origin in *E. coli*; *repA*, a plasmid-encoded gene required for *pSC101* replication; *par*, partition locus; *C. thermocellum* *pyrF* cassette; pBAS2 sequences including putative *C. bescii* replication origin; regulatory and *rho* independent terminator sequences surrounding Cbes2303; C-terminal 6X Histidine-tagged version of CelA sequences without signal peptides are indicated. All primers used in this construction are also indicated.


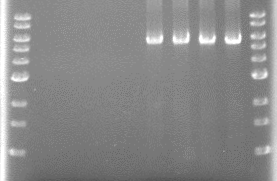


**6 kb**

**5 kb**

**Figure B. PCR confirmation of expression vector transformation.** Amplification of CelA gene on the expression vector from DNA isolated from strains. Lane 1, wildtype strain JWCB001; Lane 2, JWCB005 *ΔpyrFA;* Lane 3, JWCB018 *ΔpyrFA* *ldh::ISCbe4 Δcbe1*; Lane 4, JWCB029 *ΔpyrFA* *ldh::ISCbe4 Δcbe1 ΔcelA*; Lane 5, JWCB040 *ΔpyrFA* pDCW170::*celA* (no signal sequence); Lane 6, JWCB046 *ΔpyrFA* *ldh::ISCbe4 Δcbe1 ΔcelA* pDCW173::*celA* (with the signal sequence); and positive controls from pDCW170 (lane 7) and pDCW173 (lane 8) NEB DNA 1 kb ladder (lane 1 and 9)


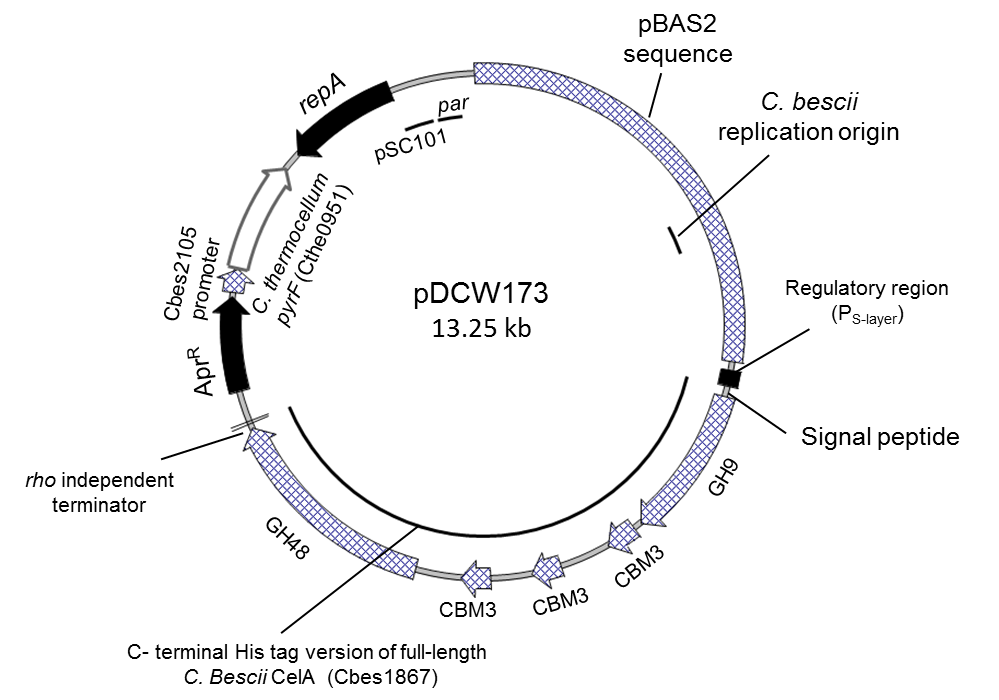


**Figure C. Diagram of the version of full-length extracellular CelA expression vector pDCW173.** ORFs from *E. coli* indicated as black arrows. Also as indicated: the apramycin resistant gene cassette (Apr^R^); pSC101, low copy replication origin in *E. coli*; *repA*, a plasmid-encoded gene required for pSC101 replication; *par*, partition locus; *C. thermocellum pyrF* cassette; pBAS2 sequences including putative *C. bescii* replication origin; regulatory and *rho* independent terminator sequences of Cbes230; C-terminal 6X Histidine-tagged version of CelA sequences with signal peptides.


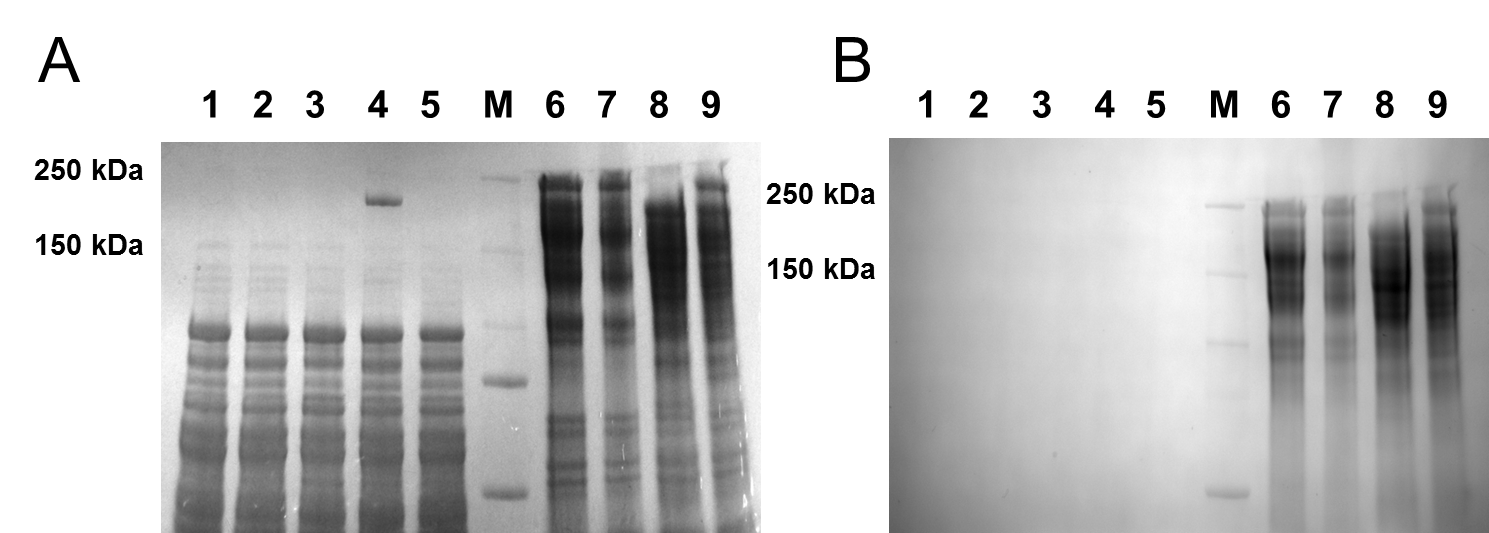


**Figure D. Evidence for extracellular glycosylation of CelA (expanded).** Panel A, Commassie stained gel. Panel B, the same gel stained with Glycoprotein Stain (G-Biosciences).Lane1, cell free extract from WT (JWCB001); Lane 2, cell free extract from JWCB005 *ΔpyrFA*; Lane 3, cell free extract from JWCB029 *ΔpyrFA* *ldh::ISCbe4 Δcbe1 ΔcelA*; Lane 4, cell free extract from JWCB040 *ΔpyrFA* pDCW170::*celA* (no signal sequence); Lane 5, cell free extract from JWCB046 *ΔpyrFA* *ldh::ISCbe4 Δcbe1 ΔcelA* pDCW173::*celA* (with the signal sequence); M, molecular weight markers (Biorad); Lane 6, supernatant from JWCB005; Lane 7, supernatant from JWCB018 *ΔpyrFA* *ldh::ISCbe4 Δcbe1*; Lane 8, supernatant from JWCB029 *ΔpyrFA* *ldh::ISCbe4 Δcbe1 ΔcelA*; Lane 9, JWCB046 *ΔpyrFA* *ldh::ISCbe4 Δcbe1 ΔcelA* pDCW173::*celA* (with the signal sequence).

**Figure E. Purification and verification of extracellular His-tagged CelA.** Panel A. Coomassie stained gel of molecular weight markers (Novex pre-stained SeeBlue Plus2) protein standard, lane 1; a well characterized His-tagged carbohydrate binding module (CBM), lane 2; purified His-tagged CelA using a HisTrap FF crude column from extracellular proteins, lane 3: CelA protein purified by HisTrap FF crude column followed by size exclusion chromotagraphy on a HL26/60 200PG. Panel B. A western of the same gel reacted with His-tag antibody.

Extracellular protein (ECP) from JWCB046 was collected from 10 L cultures grown to mid-log phase at 65°C in a fermenter with pH control, centrifuged (8,000 x g at 4°C for 10 min), filtered (glass fiber, 0.7 µm) to separate out cells, and concentrated with a 10 kDa molecular weight cut off column. The concentrated ECP was buffer exchanged with a 50 mM Tris, 100mM NaCl, 10mM imidazole solution (pH 8.0). His-tagged CelA was then extracted using a GE 5mL HisTrap FF crude column at a flow rate of 5mL/min. The resulting fraction was submitted to size exclusion chromatography on a HL26/60 200PG at a flow rate of 2mL/min using a 20mM acetate buffer containing 10mM CaCl2 and 100mM NaCl at pH 5.5. The His-tag purified protein (10-15 µg) was analyzed by SDS-PAGE (Bis-Tris NuPAGE, 4-12%) in MOPS buffer run at 200V for one hour. Protein was then transferred from the SDS-PAGE gel to a western blot membrane using an iblot blotting system for 8 minutes. The primary antibody used was an Anti-His (C-terminal) mouse monoclonal antibody (Invitrogen). The two strong bands in each of lanes 3 and 4 were analyzed using LC-MS/MS to identify the protein as CelA (Proteomics Department, Colorado State University). Larger bands likely result from glycosylation and smaller bands from protein degradation.

**Table A.** Primers used in this study.

| Primers | Sequences (5’ to 3’) | Description |
| --- | --- | --- |
| DC228 | ATCATCCCCTTTTGCTGATG | To confirm transformants |
| DC371 | AGAGCATGCTTGATTGCCAAACAGTATTTCATATGTTGC | To construct pDCW170 & 173 |
| DC460 | AGAGAGCGATCGACAGTTTGATTACAGTTTAGTCAGAGCT | To construct pDCW170 |
| DC461 | AGAAGAAGGCGGCCGCTTGGTTCCTTAAATCTAAGAGGTATGA | To construct pDCW170 |
| DC464 | ACTGGATCCCTCACCAAACCTCCTTGTATGAT | To construct pDCW170 & 173 |
| DC466 | AGAGCATGCCATCACCATCACCATCACTAATAATAAAGCTGAAATAAAAGAGGGTGAGA | To construct pDCW170 & 173 |
| DC481 | AGACTCCGATCGATTCCCATGAGCCCACGAACAGT | To construct pDCW170 |
| DC482 | AGAAGAAGGCGGCCGCTCTGACGCTCAGTGGAACGAA | To construct pDCW170 |
| DC530 | AGAGGATCCATGTCGTTTAACTATGGGGAAGCTTTACA | To construct pDCW170 |
| DC560 | AGAGGATCCATGAAGCGTTACAGAAGAATTATTGCCA | To construct pDCW173 |
| DC569 | AGAGTAGAGCGTGATGACATAGA | To confirm transformants |
